# Recent Developments and Applications of Hemicellulose From Wheat Straw: A Review

**DOI:** 10.3389/fbioe.2021.690773

**Published:** 2021-06-22

**Authors:** Ling-Zhi Huang, Ming-Guo Ma, Xing-Xiang Ji, Sun-Eun Choi, Chuanling Si

**Affiliations:** ^1^Beijing Key Laboratory of Lignocellulosic Chemistry, Research Center of Biomass Clean Utilization, Engineering Research Center of Forestry Biomass Materials and Bioenergy, College of Materials Science and Technology, Beijing Forestry University, Beijing, China; ^2^State Key Laboratory of Biobased Material and Green Papermaking, Qilu University of Technology (Shandong Academy of Sciences), Jinan, China; ^3^Department of Forest Biomaterials Engineering, College of Forest and Environmental Sciences, Kangwon National University, Chuncheon, South Korea; ^4^Tianjin Key Laboratory of Pulp and Paper, Tianjin University of Science and Technology, Tianjin, China

**Keywords:** hemicellulose, wheat straw, structure, development, applications

## Abstract

Hemicellulose is an important component of plant cell walls, which is mainly used in biofuels and bioproducts. The hemicellulose extracted from different plant sources and plant locations has different microstructure and molecule. Wheat straw is an important biomass raw material for the extraction of hemicellulose. The aims of this review are to summary the recent developments and various applications of hemicellulose from wheat straw. The microstructure and molecule of hemicellulose extracted by different methods are comparably discussed. The hemicellulose-based derivatives and composites are also reviewed. Special attention was paid to the applications of hemicellulose such as biofuel production, packaging field, and adsorbent. The problems and developing direction were given based on our knowledge. We expect that this review will put forward to the development and high-value applications of hemicellulose from wheat straw.

## Introduction

Hemicellulose can be defined as cell wall polysaccharides, which binds strongly to cellulose microfibrils by hydrogen bonds and Van der Waals force (Carvalheiro et al., 2008; Liu et al., 2020; Liu K. et al., 2021). In general, hemicellulose consisted of a heterogeneous group of plant-derived polysaccharides including D-xylose, D-mannose, D-galactose, L-arabinose, D-galactose, and 4-*O*-methyl-D-glucuronic acid. Hemicellulose is used to produce alcohol by fermentation and sorbitol by reduction (Gírio et al., 2010), which has important applications in food, toothpaste, cosmetics, explosive manufacturing, and papermaking (Falco et al., 2013; Zhao et al., 2014). Pentose of hemicellulose is also used to produce feed yeast, furoic acid, xylose, and xylitol (Yoon et al., 2006; Du et al., 2019; Liu X. et al., 2019). More importantly, xylooligosaccharides, as one of the degradation products of hemicellulose, are widely used in functional food and pharmaceutical fields due to their unique physical and chemical properties and physiological functions (Bian et al., 2014).

The hemicellulose is extracted from different plant sources and plant locations which has different structure and molecule (Peng et al., 2012; Fu et al., 2017). For example, the hemicelluloses from different biomass species such as hardwood, softwood, and gramineous plants have different chemical structures. The hemicelluloses in the secondary wall of hardwood cells are mainly glucuronoxylan or 4-*O*-methyl-glucuronoxylan with some acetyl groups; meanwhile the hemicelluloses of softwood cells are mainly galactose glucose mannan or *O*-ethyl-galactose glucose mannan (Kapu and Trajano, 2014). The structures of hemicellulose are different with different parts of the same raw material. Moreover, different raw materials and different extraction methods lead to different types and contents of side chains in the as-obtained hemicelluloses (Buranov and Mazza, 2010; Ma et al., 2012; Sun et al., 2014). Wheat straw is an important biomass raw material for the extraction of hemicellulose (Sun et al., 2005a). Recently, more attentions have been focused on the hemicellulose from wheat straw (Zhong et al., 2015).

This current review aims to describe the recent developments and various applications of hemicellulose from wheat straw, introduce the structures and molecules of hemicellulose extracted by different methods, provide the applications as biofuel production, packaging materials, and adsorbent via some typical examples about the hemicellulose-based derivatives and composites, and suggest the problems and develops direction of hemicellulose from wheat straw. We expect that this review will put forward to the development and high-value applications of hemicellulose from wheat straw.

## The Extraction and Structure of Hemicellulose From Wheat Straw

### Acid Hydrolysis Technology

Hemicellulose is the second largest component of lignocellulosic biomass. Development of efficient and low-cost extraction methods of hemicellulose is important to realize the practical applications of biomass. Dilute acid hydrolysis technology is widely used to hydrolyze hemicellulose with high conversion rate, further fermenting to produce fuel ethanol (Jiang et al., 2015). Hemicellulose can also be hydrolyzed directly to produce functional foods such as oligosaccharides and chemicals such as furfural (Luo et al., 2019). As early as 1986, in the Gonzalez’s work, a kinetic model was suggested about the hydrolysis of wheat straw hemicellulose with sulfuric acid at 34 and 90°C (Gonzalez et al., 1986). It yielded complete solubilization of hemicellulose to xylose and arabinose without significant amounts of furfural at 90°C. Authors provided a two-consecutive reaction mechanism on the kinetic model of the acid-catalyzed hydrolysis to explain the different behavior of the concentration of the two main sugars. In 2006, Canilha et al. (2006) estimated the effects of temperature and acid loading on the yield of monomeric xylose recovery from wheat straw hemicellulose. It achieved a xylitol production of 30.8 g L^–1^ after 54 h of fermentation, resulting in a productivity of 0.57 g L^–1^ h^–1^ and bioconversion yield of 0.88 g g^–1^. Authors also obtained the maximum specific rates of 0.19 g g^–1^ h^–1^ for xylose consumption and 0.15 g g^–1^ h^–1^ for xylitol production. Then, Sun’s group applied the different acid solutions of acetic acid/H_2_O, acetic acid/H_2_O, and formic acid/acetic acid/H_2_O for the treatment of the dewaxed wheat straw using 0.1% HCl as a catalyst at 85°C for 4 h, yielding 14.2–76.5% of the original hemicelluloses (Xu et al., 2006). It was found that xylose as a major constituent, glucose and arabinose in noticeable amounts, uronic acids (principally 4-*O*-methyl-D-glucuronic acid) in a small amount, and galactose, mannose, and rhamnose as minor components. They indicated that organic acids treatment induced the more linear and partially acetylated hemicelluloses with lower molecular weights ranging between 8,480 and 18,940 g mol^–1^. After that, they acetylated wheat straw hemicellulose with acetic anhydride using iodine as a catalyst in 1-butyl-3-methylimidzolium chloride [(C_4_mim)Cl] ionic liquid (Ren et al., 2007). It esterified 83% hydroxyl groups in native hemicellulose and achieved acetylated hemicellulose with yield from 70.5 to 90.8% and degree of substitution between 0.49 and 1.53 by changing the experimental parameters. The dilute sulfuric acid was also used as a catalyst for the extraction of hemicellulose with 85.5% of the total sugar from rapeseed straw at 152.6°C for 21 min (Jeong et al., 2010). The hemicellulose-derived carbohydrates were obtained from wheat straw using dilute HCl or FeCl_3_ solutions at 120°C by Marcotullio et al. (2011). It approached the recovery of 100% of xylose and arabinose. Authors found the partial precipitation of FeCl_3_ dilute solutions to iron oxides and consequent formation of HCl, facilitating the hydrolysis of hemicellulose. Obviously, acid was reported as catalyst for the extraction of hemicellulose. A solid acid SO_4_^2–^/Fe_2_O_3_ catalyst with both Lewis and Brønsted acidity was applied for the selective hydrolysis of hemicellulose from wheat straw (Zhong et al., 2015). It obtained a maximum hemicellulose hydrolysis yield of 63.5% from wheat straw at 141.97°C for 4.1 h with the ratio of wheat straw to catalyst (w/w) of 1.95:1. The catalyst was reported to be recycled six times with high activity remaining. The acidic dioxane/water solution and dimethyl sulfoxide was also reported to extract the original hemicellulose with high yield/purity from ball-milled wheat straw (Sun et al., 2005a). The toxicity of acidic dioxane don’t meet the principles of green chemistry. It obtained the arabinoxylans as the major polysaccharides, substituted by R-L-arabino furanose, 4-*O*-methylglucuronic acid, acetyl group, and xylose at *O*-3 and/or *O*-2 of xylans. Recently, the acid hydrolysis of hemicellulose containing about 30% xylan was also reported for the preparation of xylo-oligosaccharides (Wang et al., 2019). Authors found that sulfuric acid as the hydrolysis catalyst influenced the yield of xylo-oligosaccharides. The acid hydrolysis technology is a traditional process for the hydrolyze hemicellulose. However, some problems need to be solved in the near future such as the chemical reactivity, active site, and proton affinity.

### Supercritical CO_2_ Technology

CO_2_ has the advantages of non-toxic, low cost, and non-flammable (Middleton et al., 2015; Li M. G. et al., 2020). Supercritical CO_2_ can be used for the precipitation of hemicellulose due to its special physical and chemical properties at critical temperature of 31.8°C and pressure at 7.4 MPa. For example, supercritical CO_2_ as an antisolvent was applied to precipitate hemicellulose from the mixture of dimethyl sulfoxide and water by Haimer et al. (2010). The antisolvent CO_2_ was added to hemicellulose solution by single-phase transfer in supercritical state, inducing the precipitation of hemicellulose under supersaturated condition. A high-pressure CO_2_ was also used as catalyst for the selective hydrolysis of wheat straw hemicellulose on the hydrothermal production of hemicellulose-derived sugars (Relvas et al., 2015). It yielded 79.6 g of xylo-oligosaccharide per 100 g of the initial xylan content, compared with that of water (70.8 g). Authors suggested a high dissolution of wheat straw hemicellulose due to the *in situ* formation of carbonic acid by the addition of CO_2_ to water-based processes. More recently, high-pressure CO_2_-H_2_O was also reported to treat wheat straw to selectively hydrolyze hemicellulose with low concentration of acetic acid at 180°C for 1 h due to the *in situ* formation of carbonic acid and acetic acid (Yang H. P. et al., 2020). It obtained the removal ratio of 82.3% for hemicellulose. Authors suggested the high-pressure CO_2_-H_2_O as effective method in removing and hydrolyzing the hemicellulose of wheat straw. Hydrothermal CO_2_-assisted pretreatment was carried out for selective degradation of hemicellulose in wheat straw to produce pentose and enhancement the efficiency of enzymatic hydrolysis for glucose production (Wang et al., 2021). It achieved the improved efficiency of enzymatic hydrolysis and 90.0% of degraded hemicellulose at 200°C for 10 min. More than 72.7% of glucose was observed after enzymatic hydrolysis, compared with that of the untreated sample (30.2%). The hydrothermal CO_2_-assisted pretreatment was developed as an effective approach for pretreatment of biomass. In general, supercritical CO_2_-assisted pretreatment is a promising route for the extraction of hemicellulose. However, it has the disadvantages of high pressure, high lost, expensive equipment, and destroy chemical structure of hemicellulose more or less. It is necessary to balance the advantages and disadvantages of supercritical CO_2_-assisted pretreatment for the extraction of hemicellulose.

### Hydrothermal Treatment Technology

Hydrothermal treatment is to create a high temperature of 100–1,000°C and high pressure of 1–100 MPa reaction environment using water as reaction medium in a special closed vessel. In 2008, Thomsen et al. (2008) reported hydrothermal treatment of wheat straw at pilot plant scale (120–150 kg h^–1^) using a three-step reactor system aiming at high hemicellulose recovery, high cellulose digestibility, and low lignin hydrolysis. It obtained the high hemicellulose recovery (83%) at the water addition (600 kg h^–1^) at 170–180°C, meanwhile it resulted in low hemicellulose recovery (33%) and high glucose yield in the enzymatic hydrolysis with no water addition xylose degradation. The hydrolysis technology of hemicellulose was studied in corncob (Thomsen et al., 2008). It obtained the gradually increasing D-xylose and L-arabinose concentration with the growing time of repeated use of corncob hydrolyzate. Authors achieved the concentrations of D-xylose of 196.7 g⋅L^–1^ and L-arabinose of 22.0 g⋅L^–1^ after the fifth repetition. More recently, the combination of hydrothermal treatments and alkalis was also used to extract hemicellulose from de-starched corn fiber (Kaur et al., 2020). It obtained maximum yield of 47% soluble (w/w) via thermochemical treatment in the liquid fraction. It observed the compositions of extracted soluble in arabinoxylan, monosaccharides, ferulic acid, and oligosaccharides. The main difference of hydrothermal treatments lied in temperature and pressure, inducing different reaction environment and favoring the extraction of biomass. The basic mechanism of thermodynamics and kinetics of hydrothermal treatment is complex and needs to be investigated in the near future.

### Alkali Treatment Method

Besides acid hydrolysis technology, alkali method is widely used for the extraction of hemicellulose, making the cellulose swelling, breaking the bonds between hemicellulose and lignin, and dissolving the hemicellulose from the cell wall. All the type and concentration of alkali, extraction time and temperature are found to affect the yield of hemicellulose. As early as the year of 1951, Adams and Castagne (1951) did pioneering work on the alkali treatment on biomass. They first reported the various hemicellulose fractions extracted from wheat straw holocellulose with cold and hot water, and potassium hydroxide recovered by precipitation with alcohol. In their work, it found a high uronic acid and methoxyl content in the soluble fractions and a high pentosan content in the less soluble fractions. All the D-xylose, L-arabinose, D-glucose, and D-galactose were obtained. It also achieved the acid-resistant uronic acid complex of D-xylose and a monomethoxyl galacturonic acid. After that, there are many reports on the alkali treatment for hemicellulose extraction. For example, Jin et al. (2009) dissolved hemicellulose from mild ball-milled cell wall of lignified barley straw and maize stems using alkali treatments. It obtained the dissolution of the original hemicellulose of 94.6% from barley straw and of 96.4% from maize stems. Authors found the different components using different alkali solutions. The high concentration of alkali improved the isolation of hemicellulose. Sun et al. (2011) extracted the 87% of original hemicellulose from the sequential treatments of barley straw using alkali treatments. It obtained the acidic arabinxylans as the major polysaccharides, substituted by α-l-arabinofuranose, 4-*O*-methyl-glucuronic acid, acetyl group, and xylose at *O*-3 and/or *O*-2 of xylan. The cold alkaline extraction and subsequent separation by precipitation with ethanol was also developed to selective extract the hemicellulose from wheat straw (Garcia et al., 2013). It achieved the 56.1% of all hemicellulose using cold alkaline at 40°C for 90 min and recovered 39.4% of all hemicellulose by precipitation with ethanol in the raw material. The autohydrolysis and aqueous ammonia extraction of wheat straw was reported at 170–200°C by Sipponen et al. (2014). It obtained the oligomeric arabinoxylans at 66% xylan recovery yield. Authors found the autohydrolysis severity as a crucial parameter affecting properties of hemicellulose and lignin. Recently, Ragab et al. (2018) extracted hemicellulose from Egyptian agriculture wastes rice straw and husk by 4% sodium hydroxide at 90°C, purifying by 5% hydrogen peroxide, and sulfating using two catalysts of *N,N*-dicyclohexylcarbodiimide and 4-dimethylaminopyridine. The sulfated hemicellulose has the highest degree of sulfation with low total carbohydrate content, showing the promising biological activities such as anticoagulation activity at 31.25 μg mL^–1^ and fibrinolytic activity lysis more than 80% at 2,000 μg mL^–1^. During the process of alkaline treatment, it ionized the anions of OH compounds in aqueous solution, accepted protons, and provided electron donors. There are many types of organic and inorganic bases, ringing infinite opportunity for the hemicellulose extraction.

### Combination of Various Methods

Combination of various methods such as ultrasonic-assisted method, basic hydrogen peroxide method, mixed organic solvent extraction method, steam pretreatment method, microwave-assisted method, and mechanical-assisted method, maybe induce unexpected results for the extraction of biomass. Various separation and purification for wheat straw, and their advantages and disadvantages, are summarized in Table 1. In 2002, Sun et al. (2002) comparatively studied the effect of ultrasound on the yield and physiochemical properties of hemicellulose from wheat straw using 0.5 M NaOH in 60% aqueous methanol. Cavitation effect is produced by instantaneous high-temperature and high-pressure during ultrasound process, providing microenvironment for the extraction of biomass. It obtained an increasing yield of hemicellulose from 2.9 to 9.2% of the original hemicellulose for 5–35 min. The hemicellulose showed a slightly low molecular weight and slightly more linear, compared with that without ultrasonic-assisted irradiation, confirming a noticeable effect of ultrasonic irradiation. Then, 27.1–28.1% of the original hemicellulose was also extracted by the ultrasound irradiated and alkali pre-treated wheat straw with 2% H_2_O_2_-0.2% tetraacetylethylenediamine at pH 11.8 and 48°C for 12 h (Sun and Tomkinson, 2003). It obtained Xylose as a predominant sugar comprising 72.0–73.1% of the total sugars. The bleaching activator tetraacetylethylenediamine was found to form peracetic acid with hydroperoxide anion in aqueous alkali and improve the brightness of the solubilized hemicelluloses. In the existence of H_2_O_2_, it is easy to decompose into hydroxyl radical and superoxide anion radical, causing the oxidation of lignin structure, breaking the chemical bonds between lignin molecular units, and separating hemicellulose. Seven residual hemicelluloses were extracted from wheat straw pretreated with various organic solvents using 1.8% H_2_O_2_-0.18% cyanamide at 50°C and pH 10.0 for 4 h (Sun et al., 2005b). It obtained heteropolysaccharides containing xylose, glucose, arabinose, galactose, mannose, rhamnose, and 4-*O*-methyl-α-D-glucopyranosyluronic acid for the hemicellulose. It achieved the predominant monosaccharide of xylose composed mainly of L-arabino-(4-*O*-methyl-D-glucurono)-D-xylan. Cengiz et al. (2010) extracted the hemicellulose from opium poppy and cotton stalks in water at varying NaOH and H_2_O_2_. It obtained a yield of 0.8% using 2.0% H_2_O_2_ and 3.2% of the hemicellulose in the opium poppy stalks. An alkaline peroxide solution was also used to extract the hemicellulose from wheat straw, producing biodegradable hemicellulose-based films with improved water resistance and water vapor barrier properties in glycerol using citric acid as a crosslinking agent (Azeredo et al., 2015). It found the effect of the citric acid on the tensile properties of film ascribing to a flexible crosslinking.

**TABLE 1:** Summary of the separation and purification of hemicellulose from wheat straw.

**Separation and purification methods**	**Advantages**	**Limitations and disadvantages**
Acid hydrolysis technology	Effectively hydrolyze hemicellulose	Requires higher temperature and pressure
Hydrothermal treatment technology	Small structure damage; high sugar yield	High temperature
Alkali treatment method	Lower reaction temperature and pressure	Lignin dissolves more; the conversion of alkali into irrecoverable salts
Steam explosion pretreatment	Uses less chemicals; does not excessively dilute the sugar produced in the hydrolyzate; does not require excessive energy consumption	High pressure resistance of production equipment; high energy consumption
Supercritical CO_2_ technology	Non-toxic; low cost; non-flammable	High pressure; high lost; expensive equipment; destroy chemical structure of hemicellulose
Membrane separation technology	Simple to operate; the separation effect is significant	——
Graded ethanol precipitation	The simplest and most commonly used method	——

### Steam Explosion Pretreatment

Steam explosion pretreatment mainly uses high-temperature and high-pressure steam to treat biomass raw materials, which realizes the separation of components due to the explosion effect. All the time, temperature, pressure, and size of raw material have an effect on the extraction of biomass during the steam explosion process. The steam pretreatment in an alkaline environment was used to pretreat the wheat and barley straw to access high-molecular-mass hemicellulose prior to ethanol production (Persson et al., 2009). It obtained 30% of the arabinoxylan with high-molecular-mass in barley straw and more than 40% of the arabinoxylan could be extracted with high-molecular-mass in wheat straw. The steam explosion was also reported to recover hemicellulose hydrolyzates from wheat straw at 200°C for 5 min, producing microbial oil by the oleaginous fungus *Microsphaeropsis* sp. (Peng and Chen, 2012). It obtained 3.8 g L^–1^ of reducing sugar and 22.3 g L^–1^ of total soluble sugars with a 10-fold excess (w/w) of water at 40°C to wash the wheat straw. Recently, Mihiretu et al. (2019) applied steam explosion pre-treatment to extract xylan-rich biopolymers from alkali-impregnated lignocelluloses and investigated simultaneously increasing the enzymatic digestibility of cellulose. It achieved the maximum xylan yields of 51% for sugarcane trash at 204°C for 10 min. The steam explosion pre-treatment was suggested as viable biorefinery approach to co-produce xylan biopolymers and bioethanol. Based on our knowledge, steam explosion pretreatment is a promising strategy for the industrial production.

### *N,N*-Dimethylformamide–Lithium Chloride System

The *N,N*-dimethylformamide and lithium chloride system was usually applied to dissolve the cellulose. Besides, this system was also developed to treat hemicellulose (Farhat et al., 2017). Sun’s group did many works on the lauroylation of hemicellulose in a *N,N*-dimethylformamide and lithium chloride system. In 2000, 10% KOH/0.5% Na_2_B_4_O_7_⋅10 H_2_O was used to extract hemicellulose from delignified rye straw, performing esterification of the hemicelluloses with various acyl chlorides in a *N,N*-dimethylformamide and lithium chloride system using 4-(dimethylamino)pyridine catalyst and triethylamine as a neutralizer (Sun et al., 2000). It stearoylated >90% of the free hydroxyl groups in native hemicelluloses at 75°C for 40 min. Only a minimal degradation of the macromolecular hemicellulose was observed during rapid reactions at 48–75°C for 20–40 min. Then, they reported lauroylation of wheat straw hemicelluloses with lauroyl chloride and degrees of substitution ranging from 0.46 to 1.54 in *N,N*-dimethylformamide–lithium chloride using 4-dimethylaminopyridine as a catalyst (Peng et al., 2008). It observed the lauroylation preferably at the C-3 hydroxyl group of b-D-Xylp units in the hemicelluloses, and the increased thermal stability of the hydrophobic polymers by esterification. They also applied microwave-assisted method for the esterification of wheat straw hemicelluloses using *N*-bromosuccinimide as a catalyst in *N,N*-dimethylformamide–lithium chloride medium with acetyl chloride, propionyl chloride, n-octanoyl chloride, lauroyl chloride, palmitoyl chloride, stearoyl chloride, and oleoyl chloride (Xu et al., 2008). Microwave-assisted extraction is to heat biomass by electromagnetic radiation, heating the water molecules in a very short time, converting the microwave field energy into heat energy, and promoting the extraction of the main components of the biomass. It observed the esterification preferentially at the C-3 and C-2 positions. It found a partial degradation of the polymer and a slight decrease in thermal stability of the hemicellulosic derivatives by microwave irradiation. The microwave irradiation was also used for the lauroylation of wheat straw hemicellulose in the *N,N*-dimethylformamide/lithium chloride system at 78°C for 1–8 min (Ren et al., 2008). It observed the lauroylation at the C-3 position of the xylose unit in hemicelluloses. Authors indicated the high thermal stability of lauroylated polymers with high degree of substitution. Recently, the microwave-assisted hydrothermal pretreatment and subsequent alkali post-treatment was performed to isolate water- and alkali-soluble hemicellulose from hybrid pennisetum (Sun et al., 2019), which was mainly composed of β-(1→4)-linked xylans. It obtained 92.8% of the enzymatic digestibility of the cellulose-rich substrate, which was 3.4 times higher than that of raw material (27.4%). Yuan et al. (2019) also investigated microwave-assisted hydrothermal extraction of non-structural carbohydrates and hemicelluloses from tobacco biomass. It achieved the maximum yields for the leaf of 118.57 mg g^–1^ and stem of 120.33 mg g^–1^ biomass, producing the hemicelluloses yield of 105.15 mg g^–1^ at 200°C, and obtaining heterogeneous compositional type including xylan, glucuronoxylan, and xylanglucan. Obviously, combination of various methods displayed widely applications and distinct advantages over the traditional system for the extraction of biomass.

### Membrane Separation Technology

Membrane separation technologies such as microfiltration, ultrafiltration, nanofiltration, and reverse osmosis, have been widely used in polymer separation in recent years. Both ultrafiltration and nanofiltration are applied in the separation and purification of hemicellulose, which can be used to concentrate the extract and remove some low molecular weight lignin and inorganic salts. Thuvander and Jönsson (2019) used ultrafiltration to concentrate arabinoxylan isolated from wheat bran. It obtained the flux of the untreated solution of 51 L m^–1^ for 2 h, the flux after prefiltration of the solution with diatomaceous earth of 62 L m^–1^ for 2 h, and the flux of 230 L m^–1^ for 2 h after 5 h of air sparging during ultrafiltration. It achieved the retention of hemicellulose of 96% during ultrafiltration with a ceramic membrane and 93% with the prefiltered, air-sparged solution due to the size reduction of the hemicelluloses. Al-Rudainy et al. (2020) reported the recovery of hemicellulose by ultrafiltration from lab-scale to on-site pilot trials. It kept a stable average flux of 88 L m^–1^ for 2 h and the non-changing retention of products using an alkaline cleaning step (pH 11) for 1 h. Thuvander et al. (2018) used air sparging to increase the flux during ultrafiltration of alkali-extracted wheat bran hemicelluloses. Air sparging reduced the energy demand per m^3^ permeate produced during dead-end batch ultrafiltration at 80°C and 1 m/s from 0.96 kWh/m^3^ to 0.51 kWh/m^3^. They also developed air sparging of alkaline-extracted wheat bran hemicellulose prior to ultrafiltration to increase the flux (Thuvander and Jonsson, 2020). It obtained the increased average flux during ultrafiltration of 151 L m^–1^ for 2 h and during diafiltration of 130 L m^–1^ for 2 h by air sparging. The cost of purifying the hemicellulose was reduced from 1375 €/ton hemicellulose to 1122 €/ton by sparging the solution with air prior to membrane filtration.

Besides the extraction of hemicellulose from wheat straw, there are some reports on the extraction and purification of hemicellulose from other biomass. For example, sugarcane is one of the main raw materials for sugar production. In general, about 50% of the fiber of bagasse left after sugar extraction can be used for papermaking. The extraction of hemicellulose was developed by a pH pre-corrected hot water pretreatment to reduce adsorbable organichalogen formation in chlorine dioxide bleaching of bagasse pulp (Yao et al., 2017). It found the differences in the structure of the bagasse hemicelluloses and the solid residual hemicelluloses. De Oliveira Santos et al. (2018) pretreated three diverse sugarcane hybrids with dilute sulfuric acid to evaluate the role of hemicellulose removal on the efficiency of enzymatic conversion of glucan. It found the selective removal of hemicellulose with the enhanced efficiency of glucan enzymatic hydrolysis up to 63% conversion and enhanced enzymatic glucan conversions to 92–100% by post-delignification of acid-pretreatment. The purification of hemicellulose was also reported from sugarcane bagasse alkaline hydrolyzate using an aromatic-selective adsorption resin (You et al., 2019). Resin treatment had a minute effect on the molecular weight, structure, and property of hemicellulose, but could significantly improve its separation and purification. Both the chemical and structural differences were investigated in hemicellulose isolated from the sugarcane stem using chemical techniques (Yang H. Q. et al., 2020). It obtained the sugarcane hemicellulose backbone of xylose residues connected via β-1,4 glycosidic linkages, substituted with arabinose, acetyl, and glucuronic acid side chains. Manaf et al. (2018) investigated the potential usage of soluble products of oil palm frond bagasse upon dilute-acid hydrolysis. It recovered 18.4 g xylose and 8.9 g glucose per 100 g oil palm frond from 4% (v/v) HNO_3_ at 130°C for 20 min. Authors found structural changes of the oil palm frond upon acid hydrolysis, obtaining the maximum yield of 0.35 g xylitol per g of sugars using the oil palm frond hydrolyzate.

Corn straw is an important production resource for industrial and agricultural production. As a kind of feed, corn straw is rich in nutrients and available chemical components, which can be used as raw materials for animal feed. Egues et al. (2012) performed the potential of autohydrolysis and alkaline extraction processes from corn stalks for high purity hemicellulose extraction. It obtained the maximum yield of 54% of the raw material lignin-free autohydrolysis hemicellulose with the presence of sulfur as predominant contaminant in alkaline extraction. Zhang et al. (2018) studied the effect of the organizational difference of corn stalk of leaf, bark, and pith on hemicellulose extraction and biotransformation efficiency. Authors found the corn pith as more conducive to the subsequent hemicellulose extraction and enzymatic hydrolysis to produce xylo-oligosaccharides. It obtained the highest purity of xylan of 84.89% from pith, the lowest color value of 1.43 × 10^5^, and the highest hemicellulose recovery ratio of 91.03%. It achieved the enzymatic hydrolysis ratio of 40% by pith, 30% by leaf, and 20% by bark. The molecular structure of xylooligosaccharides was investigated from corn cob in a continuous flow type hydrothermal reactor (Arai et al., 2019). It found high yields and high solubility of the CX due to the acetylation and ferulic acid modification of pentose residues. He et al. (2020) analyzed the composition and structure of the different parts of corn stalk and provided models of alkali-soluble hemicellulose transportation from corn stalk. It obtained physical mass transfer-dominated process for the dissolution processes of hemicellulose from the three parts.

### Graded Ethanol Precipitation

The graded ethanol precipitation is a simple method for hemicellulose purification, in which the separated hemicellulose solution is precipitated in different concentrations of ethanol to obtain hemicellulose with different physical and chemical properties. The graded ethanol precipitation was used for the corn stalk hemicellulose from yellow liquor of active oxygen cooking (Shi et al., 2013). It observed only 5.31% hemicellulose with high molecular weight of 24,824 g mol^–1^ in the liquor and the degraded hemicellulose with low molecular weight of 2,020–4,574 g mol^–1^ during the cooking process. The hydrothermal-ethanol method was used for hemicellulose separation by the addition of 3% NaOH and 3% H_2_O_2_ (Li J. B. et al., 2020). It occurred the lignin degradation and crosslinking/polymerization in parallel both the hydrothermal treatment and ethanol extraction. The hemicellulose pyrolysis mechanism was investigated based on the functional group evolution in xylan chars between 200 and 600°C by two-dimensional correlation infrared spectroscopy (Yang H. P. et al., 2020). It observed the depolymerization and ring-opening reactions of xylan at 200°C, the decarbonylation reaction at 300–450°C, and the increased dehydrogenation and polycondensation reaction of xylan at 450–600°C.

## The Applications of Hemicelluloses From Wheat Straw

### The Xylitol and Biofuel Production of Hemicellulose

Biofuel generally refers to solid, liquid or gaseous fuels extracted from biomass, replacing gasoline and diesel made from petroleum, which is an important direction of renewable energy development and utilization. Biofuels include mainly bioethanol, biodiesel, and aviation biofuels produced from biomass resources (Liu H. Y. et al., 2021). There is no doubt about wheat straw hemicellulose for the biofuel production. As early as 1982, Detroy et al. (1982) reported bioconversion of wheat straw cellulose/hemicellulose to ethanol by *Saccharomyces uvarum* and *Pachysolen tannophilus*. It obtained the yield of cellulosic pulp of 70–82% at 170°C for 30–60 min at a water-to-solids ratio of 7:1. However, it achieved the ethanol efficiencies of only 40–60% due to the inhibition by substances introduced by thermal and alkali treatment of the straws. *Pachysolen tannophilus* strain NRRL 2460 is found to be capable of producing ethanol from both glucose and xylose. In 2001, Nigam evaluated the ethanol production from wheat straw hemicellulose acid hydrolyzate by boiling and over-liming with Ca(OH)_2_ using an adapted and parent strain of *Pichia stipitis*. NRRL Y-7124 (Nigam, 2001). It achieved 2.4 folds of ethanol yield and 5.7 folds of productivity, compared to neutralized hydrolyzate. It obtained the maximum yield of 0.41 g_*p*_ g_*s*_^–1^, equivalent to 80.4% theoretical conversion efficiency. The acetic acid, furfurals, and lignin were inhibitory to microbial growth and ethanol production, resulted a reduction in ethanol yield and productivity.

**FIGURE 1:**
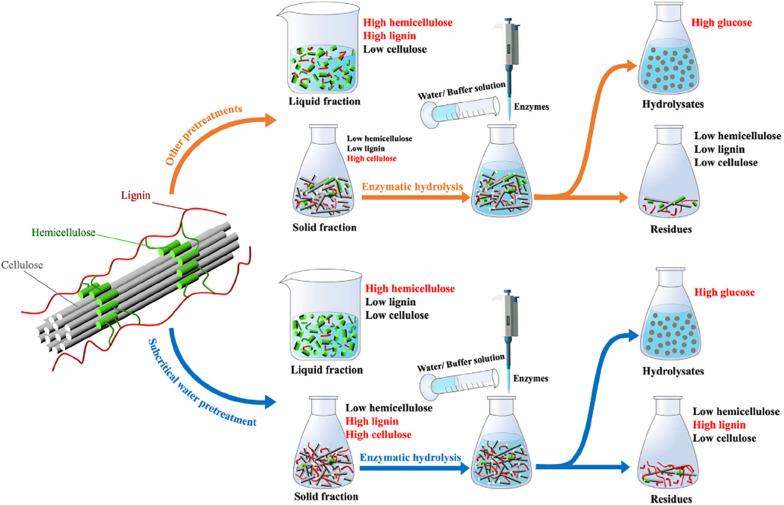
Different variations of lignin and cellulose content between subcritical water pretreatment and other pretreatments (Chen et al., 2021).

Koti et al. (2016) reported the enhanced bioethanol production from wheat straw hemicellulose by mutant strains of pentose fermenting organisms *Pichia stipitis* and *Candida shehatae*. It obtained the enhanced ethanol production of 12.15 ± 0.57 g L^–1^ and yield of 0.450 ± 0.009 g g^–1^, compared with that of the wild strains (8.28 ± 0.54 g L^–1^ and yield 0.380 ± 0.006 g g^–1^). In their work, the excellent points are about the stable of mutant strains for 19 cycles in hemicellulosic hydrolyzates of wheat straw, and the combination of chemical mutagenesis and UV induced mutants. Tsegaye et al. (2019) investigated microwave-assisted NaOH pretreatment of wheat straw using central composite design under varying operating variables of pretreatment time, temperature, and NaOH concentration. It preserved 38.34% of hemicellulose and 74.15% of cellulose in the solid residue and solubilized 69.49% of lignin. It released 718 mg g^–1^ of reducing sugar after hydrolysis pretreated wheat straw by *Bacillus* sp. *BMP01* and obtained ethanol yield of 68.2% after 96 h of fermentation due to the combination of microwave-assisted NaOH pretreatment coupled with microbial hydrolysis and C5 and C6 fermenting microbes. Then, they optimized organosolv pretreatment parameters for lignin removal and polysaccharide release using response surface methodology by microbial hydrolysis for biofuel production (Tsegaye et al., 2020). It solubilized 73.17% of lignin and 46.62% hemicellulose, and released 74.09% of cellulose at 75.4°C for about 30 min. The enhanced sugar and ethanol yields were due to the combination of organosolv pretreatment and *Bacillus* sp. *BMP01* hydrolysis of rice straw. The subcritical water pretreatment and high solid hydrolysis were used to improve the conversion efficiency of bioethanol from wheat straw (Chen et al., 2021), as shown in Figure 1. It carried out the yields of hydrolysis of 77.85–89.59% and fermentation of 93.34–96.18%. It observed the improved ethanol concentration of 37.00 g L^–1^. Authors indicated the subcritical water pretreatment combined with high solid hydrolysis as an effective solution for bioethanol conversion.

Besides wheat straw hemicellulose, the hemicellulose from other biomass was also applied for ethanol production. Tabañag et al. (2018) reported ethanol production from hemicellulose by a consortium of different genetically modified sacharomyces cerevisiae. It observed the improved hydrolytic activities of the hemicellulase mixtures by displaying the hemicellulase on the yeast surface as whole-cell biocatalysts. The resulting consortium of hemicellulase mixtures was found to grow and produce ethanol from different xylan substrates. Scordia et al. (2012) evaluated the production of ethanol by *Scheffersomyces (Pichia) stipitis CBS6054* from sugars contained in the giant reed (Arundo donax L.) hemicellulosic hydrolyzate. Authors found the improved fermentability of the giant reed hemicellulose hydrolyzate due to the increasing initial pH from 5.0 to 6.5. It obtained 8.20 g L^–1^ of ethanol at pH 6.0 after 48 h with an ethanol yield of 0.33 (ge/gs) and a productivity of 0.17 g L^–1^ h^–1^. Mihiretu et al. (2017) investigated the viability of microwave-induced pressurized hot water conditions for co-production of xylan-based biopolymers and bioethanol from aspen wood sawdust and sugarcane trash. It obtained maximum xylan extraction yields of 66% for aspen wood and 50% for sugarcane trash. Authors found the xylan extracts predominantly in non-monomeric form. Batog et al. (2020) reported bioethanol production from biomass of selected sorghum varieties cultivated as main and second crop. It recommended the Sucrosorgo 506 variety for the production of bioethanol both in main and second crop cultivation. Sharma et al. (2019) reported the ethanol production from rice straw with 1% NaOH by autoclaving at 121°C for 30 min at 10% solid loading. It obtained 2 and 4 g L^–1^ ethanol with fermentation efficiency 55–66% fermented by *S. cerevisiae* LN for 24 h.

There are a few reports on the other biofuel production of hemicellulose. Hydrotropic reagent sodium xylene sulfonate was used to treat wheat straw for biobutanol production (Qi et al., 2019). It obtained butanol production with 12.41 g L^–1^ ABE produced by *C. acetobutylicum* using hexoses and pentoses in the enzymatic hydrolyzates. Roberto et al. (1996) evaluated the effect of inoculum level on xylitol production by *Candida guilliermondii* in a rice straw hemicellulose hydrolyzate. It reached the maximum xylitol yield of 0.71 g g^–1^ and volumetric productivity of 0.56 g L^–1^ h^–1^ with an inoculum level of 0.9 g L^–1^. It obtained the xylitol from rice straw hemicellulose hydrolyzate by the yeast *C. guilliermondii* with efficiency values as high as 77% of the theoretical maximum. Terrone et al. (2018) investigated the xylanase production by *Penicilliumchrysogenum F-15 strain* using biomass as substrate. It observed the low enzymatic activity on commercial xylan of xylanase, compared with that on hemicellulose from agroindustry biomass. In the review article, Luo et al. (2019) summarized an overview of the production of furfural directly from hemicellulose in lignocellulosic biomass with special emphasis on achieving the effective utilization of hemicellulose, including the selective dissolution of hemicellulose from lignocellulosic biomass and the selective formation of furfural from hemicellulose derivatives. They considered solvents and catalysts as two main factors in this valorization process of hemicellulose. Gao et al. (2020) investigated the pyrolysis behavior of xylan-based hemicellulose in a fixed bed reactor. It found the increase xylan conversion and yield of bio-oil at high temperature due to the violent decomposition of xylan. Shi et al. (2021) relieved the inhibition of high concentration of alkali on xylose production from hydrolysis of hemicellulose in PIE. It observed the decreased polymerization degree of the hemicellulose by 73.4%, promoting the subsequent enzymatic hydrolysis process. Authors obtained the xylose yield followed by enzymatic hydrolysis of 57.15 g L^–1^, which was 145.38% more than that of enzymatic hydrolysis alone.

### The Application of Hemicellulose-Materials in Packaging Field

Packaging materials refer to the materials used to meet the requirements of product packaging, including metal, plastic, paper, natural fiber, chemical fiber, and composite materials. Hemicellulose was expected to be used packaging field due to their hydrophilic nature, biodegradable, and low-cost. In the review paper by Farhat et al. (2017), they ascribed the processing and applications of water-resistant hemicellulose-based films and composites. They summarized the most useful pathways to change the hydrophilic character of hemicelluloses to hydrophobic and discussed several applications of these materials. The biodegradable films were formed by using wheat straw hemicelluloses as a matrix with cellulose nanocrystals and citric acid (Pereira et al., 2017). It obtained the improved tensile strength and modulus, water resistance and water vapor barrier with the addition of cellulose nanocrystals, and the plasticizing and crosslinking effects by citric acid due to a crosslinking extension by glycerol. Authors suggested the films with enhanced modulus, elongation, water resistance, and barrier to water vapor for wrapping or coating a variety of foods. In the work by Ma et al. (2018), the potential of an acid hydro-tropic process was evaluated at low temperatures for on-farm valorization of wheat straw by producing ligocellulosic nanofibrils, lignin nanoparticles, and furfural. It obtained wheat straw films with excellent mechanical properties and specific tensile strength over 120 kN.m kg^–1^. The addition of inorganic and/or inorganic materials into hemicellulose matrix is important to improve its properties. Recently, Rao et al. (2019) fabricated the hemicellulose films with enhanced mechanical properties by graphene oxide for bio-medicine, packaging materials, and humidity sensing. It obtained a high tensile strength of 43.83 MPa due to that addition of graphene oxide and high sensitivity to humidity. The storage modulus of the hybrid films had an order of magnitude change in value at different humidities. Lucenius et al. (2019) researched the hemicellulose-cellulose nanofibrils films for the packaging and medical applications to understand the interactions between the components at nano/microscale affect macroscopic mechanical properties of toughness and strength. It reported the decreased aggregates of cellulose nanofibrils and the enhanced strength of dry films due to the adsorbed polysaccharides and a moderate reduction in friction between cellulose surfaces. It obtained the improved mechanical properties of composites due to high affinity for cellulose and moderate hydration in wet conditions.

The extraction step and treatment methods played an important role in the properties of films. Jin et al. (2019) investigated the factors affecting the film-forming properties of the originally isolated xylan-hemicelluloses and promoting their material properties from the extraction step. It improved tensile strength of films up to 52% using sodium hypochlorite solution as the delignification agents. Authors predicted better mechanical properties due to hemicellulose with higher molecular weight, more linear structure, and lower lignin content. Hu et al. (2019) fabricated polysaccharide composite film from wheat straw hemicellulose and methylcellulose for packaging. It obtained films with compact layer structure and the maximum tensile strengths using 75 wt% methylcellulose. Mugwagwa and Chimphango (2020) obtained the enhanced hemicellulose acetylation, filler compatibility, and film hydrophobicity by optimizing alkali-catalyzed organosolv treatment conditions for wheat straw before hemicellulose extraction. It obtained water contact angle of 68.1° for the films. The acetylated nanocellulose reinforced films had high tensile strength of 10.59 MPa and Young modulus of 590.15 MPa.

**FIGURE 2:**
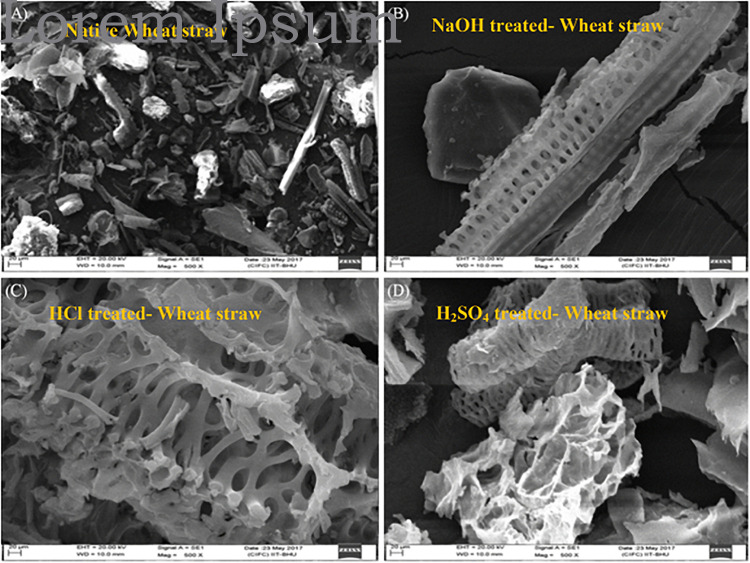
Scanning electron microscopy micrographs of native wheat straw and all pre-treated wheat straw biomass: **(A)** native wheat straw, **(B)** NaOH-treated wheat straw, **(C)** HCl-treated wheat straw, **(D)** H_2_SO_4_-treated wheat straw (Dixit and Yadav, 2020).

The multifunctional films could open the new applications of wheat straw. Li J. et al. (2019) proposed an approach to directly and completely convert natural wheat straw into multifunctional films with high mechanical strength by introducing an entanglement network of additional cellulose to enhance the strength of the regenerated straw in the ionic liquid 1-allyl-3-methylimidazolium chloride (AmimCl). It observed the spinnability and film-forming properties based on an increase in the capillary break-up time. It obtained a tensile strength of 62 MPa, a superhigh haze of 97%, preventing 97% UVA (320–400 nm), and almost 100% UVB (280–320 nm) for the films. The high-strength, high-haze, and UV-shielding all-biomass films have great potential in low-cost, biodegradable, and environmentally friendly packaging. More recently, Zhao et al. (2020) reviewed research progress in development of hemicellulose film with regard to application in the field of food packaging due to its combination of such advantages as abundance, biodegradability, and renewability. They presented the mechanical, barrier properties, and hydrophobicity for food packing materials by various physical and chemical modification approaches. Dixit and Yadav (2020) comparative studied the polystyrene/chemically modified wheat straw composite in improving surface morphology using solution casting method for packaging application. It observed the changes in crystalline structure, hydrophobicity, water vapor migration rate, and thermal and mechanical stabilities of bio-composites. There existed some impurities such as residual lignin in the surface of native wheat straw, irregular shape after applying the HCl and H_2_SO_4_ pre-treatments, and a cylindrical shape with filaments, cells, and pores after NaOH pre-treatment (Figure 2). Authors demonstrated the alkali-treated wheat straw to synthesize a biodegradable composite film for various industrial packaging application.

In general, development of transparent thin packaging materials with low-cost, non-toxic, high buffering strength, good retraction rate, puncture resistant, and tear resistance are important for their industrial applications. The wheat straw hemicellulose composite films may be a promising candidate for this purpose. The increased enhanced mechanical properties and packaging properties still need to be improved in the next work.

### The Application of Hemicellulose-Materials as Adsorbent

Batzias et al. (2009) investigated the simulation of batch and column kinetics of methylene blue and red basic 22 adsorption on mild acid hydrolyzed wheat straw as a low-cost adsorbent for wastewater dye removal. It achieved the enhanced adsorption properties of the original material by the mild acid hydrolysis, attributing to the removal of the hemicelluloses during sulfuric acid treatment, resulting in the open pores of lignocellulosic matrix’s structure and the increasing of the BET surface area. The hydrophobic hemicelluloses with the degree of substitution from 0.09 to 0.35 were prepared by the benzylation of wheat straw hemicelluloses under the presence of catalyst in an ethanol/water system (Ren et al., 2012). It observed the increased thermal stability and the hydrophobicity after the modification of hemicelluloses due to the introduction of benzyl groups. The pH-responsive hydrogels based on hemicellulose of wheat straw were prepared as a carrier for controlled drug delivery (Sun et al., 2013). It followed the swelling kinetics of the hydrogels for a Fickian diffusion process. The biodegradability of the hemicellulose-based hydrogels was affected by hemicellulose content and the crosslinking density. Both the hydrogel and the intrinsic character of the drug controlled the drug release. A stimuli-responsive porous hydrogels were synthesized from wheat straw hemicellulose using CaCO_3_ as the porogen for the removal of methylene blue (Sun et al., 2013, 2015a). The porous hydrogels showed the sensitivity to pH and salt, exhibiting rapid shrinking in NaCl aqueous solutions. Authors demonstrated the adsorption data fitted to the pseudo-first-order, pseudo-second-order, and intra-particle diffusion kinetics models for the adsorption process. The sxylan/poly(acrylic acid) magnetic nanocomposite hydrogel adsorbent with semi-interpenetrating network structure was prepared from wheat straw xylan and Fe_3_O_4_ nanoparticles for methylene blue removal (Sun et al., 2015b). It observed a macroporous structure with interconnected porous channels. It observed the removal percentage above 90%, the adsorption isotherm of the Langmuir model, and the pseudo-second-order kinetic model of the adsorption process.

The glow discharge electrolysis plasma was used to prepare the temperature/pH dual sensitivity reed hemicellulose-based hydrogels with high deswelling ratio (Zhang et al., 2015). It found the phase transition temperatures of all approximately 33°C and the deswelling dynamics of the first model. A superabsorbent hemicelluloses-g-AA/bentonite/polyvinyl alcohol hydrogel with the enhanced swelling properties was fabricated by using waste hemicelluloses lye and polyvinyl alcohol (Liu et al., 2019). A dialdehyde hemicellulose-based chitosan-Fe_3_O_4_ composite aerogel with magnetism was synthesized to remove the Congo red, extracted from straw with NaIO_4_ (Guan et al., 2020). The hydrogels were prepared by the Schiff’s base reaction, processed to obtain aerogels by vacuum freeze-drying technique. The addition of Fe_3_O_4_ was reported to improve the thermal stability, mechanical properties, and adsorption property of the aerogels. It obtained the maximum compress strength of aerogel of 0.37 MPa, and the maximum absorption capacity of Congo red dye of 137.74 mg g^–1^. Nascimento and Neto (2021) reported hydrothermal pretreatment steam explosion in the production of an adsorbent material. It observed the adsorption of the monocomponent Cu^2+^ and Cd^2+^ at room temperature due to the increase of biomass crystallinity and porosity. It found the kinetic pseudo-second-order model and the Langmuir model isotherm for the adsorption process with a maximum adsorption capacity of Cu^2+^ of 18.86 mg g^–1^ and Cd^2+^ of 17.9 mg g^–1^. da Silva et al. (2020) investigated the methylene blue adsorption by ryegrass straw as adsorbent materials. It obtained the biochar with a well-developed porous structure. It found the kinetic studies, the pseudo-first-order, pseudo-second-order, and Avrami models for the adsorption process. It obtained the maximum adsorption capacity to the milled straw of 28.7 mg g^–1^ and treated straw of 67.19 mg g^–1^ by the Sips model. Authors achieved the high efficiency in the removal of the methylene blue dye higher than 99% using the treated straw.

### The Other Applications of Hemicellulose-Materials

Ghaffar et al. (2015) reported bioengineering for utilization and bioconversion of straw biomass into bio-products of bioethanol, biogas, and bio-composites. They paid attention to the applications and limitations of biological pre-treatment in combination with mild chemical and or physical pre-treatments. Wheat straw-polypropylene composites were formed by mixing compression molding to evaluate the susceptibility to mold fungi colonization (He et al., 2016). It found the degraded hemicellulose, followed by lignin, and then cellulose via fungi. The color change was relative to carbonyl index and the process of degradation in wheat straw. Tian et al. (2018) reported the pretreatment and bioconversion of lignocellulosic biomass from wheat straw materials. They summarized the application fields, main pretreatment processing methods, and bioproducts of bioethanol, biohydrogen, and bio-composites of wheat straw. The hemicellulose was subjected to a chemical modification by ring-opening graft polymerization of ε-caprolactone to improve its processability for the bioplastic industry (Farhat et al., 2018). It synthesized hemicellulose-graft-poly-(ε-caprolactone) copolymers with a biodegradation of 95.3–99.7% using 1,5,7-triazabicyclodecene as an organic catalyst. It observed the enhanced mechanical and the hydrophobic properties by poly-(ε-caprolactone) grafting onto hemicellulose. Castillo et al. (2020) investigated the effects and mineral/reagent interactions of hemicelluloses monosaccharides of D-xylose, D-mannose, and D-glucose on the flotation behavior of molybdenite. Authors indicated the strong depressing effects of D-mannose and D-glucose due to more carbon atoms and hydroxyl groups in their structure, providing more chances to interact with the metallic sites existing on molybdenite surfaces. It found the increase depressing effect of the monosaccharides with pH by the increase of the concentration of basic sites on molybdenite surfaces and by ionization of the hydroxyl groups of monosaccharide molecules. It observed the reduced depressing effect of the monosaccharides by the addition of kerosene.

## Conclusion

In summary, there are many reports on the extractions and applications of hemicellulose from wheat straw. Rapid progress had paid on the developments of wheat straw hemicellulose. The extraction methods played an important role in the structures and molecules of hemicellulose. The different extraction methods of hemicellulose were compared and analyzed in this article. The structures and molecules of the hemicellulose obtained by different extraction methods were different. The wheat straw hemicellulose-based derivatives and composites have widely applications as biofuel production, packaging materials, and adsorbent. Hemicellulose can be converted into biofuel production by hydrolysis reaction. Hemicellulose can be used for packaging materials due to its advantages of hydrophilicity, biodegradability, and low cost. Based on our knowledge, there are some problems need to be solved: (1) Further exploring the structure-properties relationship and mechanism of hemicellulose and its derivatives; (2) Developing the new methods such as fluorescent labeling technology to study the distribution of hemicellulose in the cell wall of different raw materials and the dissolution of hemicellulose in the process of separation and extraction, so as to build a more efficient hemicellulose separation system; (3) Finding a low-cost purification process for hemicellulose and realizing industrial production, and further combining various separation and purification technologies to realize the hierarchical purification of hemicellulose; (4) Paying more attention to the separation and purification of hemicelluloses with different structures to obtain hemicelluloses with different biological and physiological activities; (5) Developing new ways of hemicellulose functionalization, optimizing the existing chemical modification system, and further studying the homogeneous derivatization of hemicellulose in various fully soluble systems and exploring its reaction mechanism; (6) Designing the structure and function of hemicellulose derivatives by molecular modification, and exploring the structure, physicochemical properties, and potential applications (especially in biomedicine and pharmaceutical chemistry) of hemicellulose derivatives to provide theoretical basis for their promotion and applications; (7) Constructing new hemicellulose functional materials at the molecular level and synthesizing the nano-hemicellulose such as hemicellulose nanoparticles and hemicellulose nanomaterials. The nano-hemicellulose was currently used in a variety of biomedical, food or food packaging, energy and environmental applications due to their enhanced bioavailability, biocompatibility, bioactivity, and lower toxicity to healthy cells and the environment; (8) Finding an effective way to increase the strength and hydrophilicity of hemicellulose membrane; (9) Exploring the biomedical properties of hemicellulose and its derivatives, such as cytotoxicity, metabolic degradation characteristics, environmental response of different parts of the body, drug and gene binding properties, etc. In essence, hemicellulose is closely attached to the surface of cellulose by hydrogen bond and van der Waals force and connects lignin by ferulic acid. The research on hemicellulose favored the applications of cellulose and lignin in the biomass. Therefore, the developments of hemicellulose not only open its new applications, but accelerate the approaches of utilization for industrial hemicellulose.

## Author Contributions

L-ZH and M-GM: investigation and writing—original draft. X-XJ, S-EC, and CS: supervision. M-GM, X-XJ, S-EC, and CS: writing—review and editing. All authors contributed to the article and approved the submitted version.

## Conflict of Interest

The authors declare that the research was conducted in the absence of any commercial or financial relationships that could be construed as a potential conflict of interest.

## References

[B1] Adams G. A. Castagne A. E. (1951). Hemicelluloses of wheat straw. *Can. J. Chem.* 29 109–122. 10.1139/v51-015 14821842

[B2] Al-Rudainy B. Galbe M. Wallberg O. (2020). From lab-scale to on-site pilot trials for the recovery of hemicellulose by ultrafiltration: experimental and theoretical evaluations. *Sep. Purif. Technol.* 250:117187. 10.1016/j.seppur.2020.117187

[B3] Arai T. Biely P. Uhliarikova I. Sato N. Makishima S. Mizuno M. (2019). Structural characterization of hemicellulose released from corn cob in continuous flow type hydrothermal reactor. *J. Biosci. Bioeng.* 127 222–230. 10.1016/j.jbiosc.2018.07.016 30143337

[B4] Azeredo H. M. C. Kontou-Vrettou C. Moates G. K. Wellner N. Cross K. Pereira P. H. F. (2015). Wheat straw hemicellulose films as affected by citric acid. *Food Hydrocoll.* 50 1–6. 10.1016/j.foodhyd.2015.04.005

[B5] Batog J. Frankowski J. Wawro A. Lacka A. (2020). Bioethanol production from biomass of selected sorghum varieties cultivated as main and second crop. *Energies* 13:6291. 10.3390/en13236291

[B6] Batzias F. Sidiras D. Schroeder E. Weber C. (2009). Simulation of dye adsorption on hydrolyzed wheat straw in batch and fixed-bed systems. *Chem. Eng. J.* 148 459–472. 10.1016/j.cej.2008.09.025

[B7] Bian J. Peng P. Peng F. Xiao X. Xu F. Sun R. C. (2014). Microwave-assisted acid hydrolysis to produce xylooligosaccharides from sugarcane bagasse hemicelluloses. *Food Chem.* 156 7–13. 10.1016/j.foodchem.2014.01.112 24629931

[B8] Buranov A. U. Mazza G. (2010). Extraction and characterization of hemicelluloses from flax shives by different methods. *Carbohydr. Polym.* 79 17–25. 10.1016/j.carbpol.2009.06.014

[B9] Canilha L. Carvalho W. Almeida e Silva J. B. (2006). Xylitol bioproduction from wheat straw: hemicellulose hydrolysis and hydrolyzate fermentation. *J. Sci. Food Agric.* 86 1371–1376. 10.1002/jsfa.2524

[B10] Carvalheiro F. Duarte L. C. Girio F. M. (2008). Hemicellulose biorefineries: a review on biomass pretreatments. *J. Sci. Ind. Res. India* 67 849–864.

[B11] Castillo I. Gutierrez L. Hernandez V. Diaz E. Ramirez A. (2020). Hemicelluloses monosaccharides and their effect on molybdenite flotation. *Powder Technol.* 373 758–764. 10.1016/j.powtec.2020.07.032

[B12] Cengiz M. Dincturk O. D. Sahin H. T. (2010). Fractional extraction and structural characterization of opium poppy and cotton stalks hemicelluloses. *Pharmacogn. Mag.* 6 315–319. 10.4103/0973-1296.71798 21120035 PMC2992146

[B13] Chen J. Wang X. Zhang B. Yang Y. Song Y. Zhang F. (2021). Integrating enzymatic hydrolysis into subcritical water pretreatment optimization for bioethanol production from wheat straw. *Sci. Total Environ.* 770:145321. 10.1016/j.scitotenv.2021.145321 33515886

[B14] da Silva E. O. dos Santos V. D. de Araujo E. B. Guterres F. P. Zottis R. Flores W. H. (2020). Removal of methylene blue from aqueous solution by ryegrass straw. *Int. J. Environ. Sci. Technol.* 17 3723–3740. 10.1007/s13762-020-02718-9

[B15] De Oliveira Santos V. T. Siqueira G. Ferreira Milagres A. M. Ferraz A. (2018). Role of hemicellulose removal during dilute acid pretreatment on the cellulose accessibility and enzymatic hydrolysis of compositionally diverse sugarcane hybrids. *Ind. Crops Prod.* 111 722–730. 10.1016/j.indcrop.2017.11.053

[B16] Detroy R. W. Cunningham R. L. Bothast R. J. Bagby M. O. Herman A. (1982). Bioconversion of wheat straw cellulose/hemicellulose to ethanol by *Saccharomyces uvarum* and Pachysolen tannophilus. *Biotechnol. Bioeng.* 24 1105–1113. 10.1002/bit.260240507 18546403

[B17] Dixit S. Yadav V. L. (2020). Comparative study of polystyrene/chemically modified wheat straw composite for green packaging application. *Polym. Bull.* 77 1307–1326. 10.1007/s00289-019-02804-0

[B18] Du H. Liu W. Zhang M. Si C. Zhang X. Li B. (2019). Cellulose nanocrystals and cellulose nanofibrils based hydrogels for biomedical applications. *Carbohydr. Polym.* 209 130–144. 10.1016/j.carbpol.2019.01.020 30732792

[B19] Egues I. Sanchez C. Mondragon I. Labidi J. (2012). Effect of alkaline and autohydrolysis processes on the purity of obtained hemicelluloses from corn stalks. *Bioresour. Technol.* 103 239–248. 10.1016/j.biortech.2011.09.139 22029960

[B20] Falco C. Sieben J. M. Brun N. Sevilla M. van der Mauelen T. Morallon E. (2013). Hydrothermal carbons from hemicellulose-derived aqueous hydrolysis products as electrode materials for supercapacitors. *Chemsuschem* 6 374–382. 10.1002/cssc.201200817 23319452

[B21] Farhat W. Venditti R. A. Hubbe M. Taha M. Becquart F. Ayoub A. (2017). A review of water-resistant hemicellulose-based materials: processing and applications. *Chemsuschem* 10 305–323. 10.1002/cssc.201601047 28029233

[B22] Farhat W. Venditti R. Ayoub A. Prochazka F. Fernandez-de-Alba C. Mignard N. (2018). Towards thermoplastic hemicellulose: chemistry and characteristics of poly-(epsilon-caprolactone) grafting onto hemicellulose backbones. *Mater. Des.* 153 298–307. 10.1016/j.matdes.2018.05.013

[B23] Fu L. H. Liu S. Li S. L. Li Y. Y. Ma M. G. (2017). Isolation and characterization of hemicelluloses by hydrothermal method with ethanol from Populus tomenttosa Carr. *Paper Biomater.* 2 1–11.

[B24] Gao Z. X. Li N. Wang Y. Q. Niu W. S. Yi W. M. (2020). Pyrolysis behavior of xylan-based hemicellulose in a fixed bed reactor. *J. Anal. Appl. Pyrolysis* 146:104772. 10.1016/j.jaap.2020.104772

[B25] Garcia J. C. Diaz M. J. Garcia M. T. Feria M. J. Gomez D. M. Lopez F. (2013). Search for optimum conditions of wheat straw hemicelluloses cold alkaline extraction process. *Biochem. Eng. J.* 71 127–133. 10.1016/j.bej.2012.12.008

[B26] Ghaffar S. H. Fan M. McVicar B. (2015). Bioengineering for utilisation and bioconversion of straw biomass into bio-products. *Ind. Crops Prod.* 77 262–274. 10.1016/j.indcrop.2015.08.060

[B27] Gírio F. Fonseca C. Carvalheiro F. Duarte L. C. Marques S. Bogel-Lukasik R. (2010). Hemicelluloses for fuel ethanol: a review. *Bioresour. Technol.* 101 4775–4800. 10.1016/j.biortech.2010.01.088 20171088

[B28] Gonzalez G. Lopez-Santin J. Caminal G. Sola C. (1986). Dilute acid hydrolysis of wheat straw hemicellulose at moderate temperature: a simplified kinetic model. *Biotechnol. Bioeng.* 28 288–293. 10.1002/bit.260280219 18555326

[B29] Guan Y. Rao J. Wu Y. L. Gao H. Liu S. Q. Chen G. G. (2020). Hemicelluloses-based magnetic aerogel as an efficient adsorbent for Congo red. *Int. J. Biol. Macromol.* 155 369–375. 10.1016/j.ijbiomac.2020.03.231 32240739

[B30] Haimer E. Wendland M. Potthast A. Henniges U. Rosenau T. Liebner F. (2010). Controlled precipitation and purification of hemicellulose from DMSO and DMSO/water mixtures by carbon dioxide as anti-solvent. *J. Supercrit. Fluids* 53 121–130. 10.1016/j.supflu.2010.02.009

[B31] He C. Yao X. Xue J. Xiong J. Zhao L. (2016). Influences of mold fungi colonization on wheat straw-polypropylene composites. *For. Prod. J.* 66 472–479. 10.13073/FPJ-D-15-00004

[B32] He L. Yang S. B. Chen D. Peng L. C. Liu Y. X. Guan Q. Q. (2020). Hemicellulose transportation from different tissues of corn stalk to alkaline hydrogen peroxide solution. *Cellulose* 27 4255–4269. 10.1007/s10570-020-03088-8

[B33] Hu G. C. Fu S. Y. Chu F. Q. Wu G. Y. (2019). Fabrication of an all-polysaccharide composite film from hemicellulose and methylcellulose. *Bioresources* 14 6716–6726. 10.15376/biores.14.3.6716-6726

[B34] Jeong T. S. Um B. H. Kim J. S. Oh K. K. (2010). Optimizing dilute-acid pretreatment of rapeseed straw for extraction of hemicellulose. *Appl. Biochem. Biotech.* 161 22–33. 10.1007/s12010-009-8898-z 20087686

[B35] Jiang L. Q. Zheng A. Q. Zhao Z. L. He F. Li H. B. Liu W. G. (2015). Obtaining fermentable sugars by dilute acid hydrolysis of hemicellulose and fast pyrolysis of cellulose. *Bioresour. Technol.* 182 364–367. 10.1016/j.biortech.2015.01.032 25690683

[B36] Jin A. X. Ren J. L. Peng F. Xu F. Zhou G. Y. Sun R. C. (2009). Comparative characterization of degraded and non-degradative hemicelluloses from barley straw and maize stems: composition, structure, and thermal properties. *Carbohyd. Polym.* 78 609–619. 10.1016/j.carbpol.2009.05.024

[B37] Jin A. X. Ren J. L. Peng F. Xu F. Zhou G. Y. Sun R. C. (2019). Promoting the material properties of xylan-type hemicelluloses from the extraction step. *Carbohydr. Polym.* 215 235–245. 10.1016/j.carbpol.2019.03.092 30981350

[B38] Kapu N. S. Trajano H. L. (2014). Review of hemicellulose hydrolysis in softwoods and bamboo. *Biofuels Bioprod. Bior.* 8 857–870. 10.1002/bbb.1517

[B39] Kaur D. Singla G. Singh U. Krishania M. J. (2020). Efficient process engineering for extraction of hemicellulose from corn fiber and its characterization. *Carbohydr. Polym. Technol. Appl.* 1:100011. 10.1016/j.carpta.2020.100011

[B40] Koti S. Govumoni S. P. Gentela J. Rao L. V. (2016). Enhanced bioethanol production from wheat straw hemicellulose by mutant strains of pentose fermenting organisms *Pichia stipitis* and Candida shehatae. *Springerplus* 5:1545. 10.1186/s40064-016-3222-1 27652118 PMC5020006

[B41] Li J. B. Feng P. Xiu H. J. Zhang M. Y. Li J. Y. Du M. (2020). Wheat straw components fractionation, with efficient delignification, by hydrothermal treatment followed by facilitated ethanol extraction. *Bioresour. Technol.* 316:123882. 10.1016/j.biortech.2020.123882 32739576

[B42] Li J. Y. Zhang X. C. Zhang J. M. Mi Q. Y. Jia F. W. Wu J. (2019). Direct and complete utilization of agricultural straw to fabricate all-biomass films with high-strength, high-haze and UV-shielding properties. *Carbohydr. Polym.* 223:115057. 10.1016/j.carbpol.2019.115057 31427002

[B43] Li M. G. Yang X. M. Lu T. L. Zhou L. P. (2020). Selective hydrolysis of hemicellulose component of wheat straw in high-pressure CO2 and water with low concentration of acetic acid. *J. Chem. Technol. Biot.* 95 2237–2242. 10.1002/jctb.6411

[B44] Li X. Xu R. Yang J. Nie S. Liu D. Liu Y. (2019). Production of 5-hydroxymethylfurfural and levulinic acid from lignocellulosic biomass and catalytic upgradation. *Ind. Crops Prod.* 130 184–197. 10.1016/j.indcrop.2018.12.082

[B45] Liu H. Y. Xu T. Liu K. Zhang M. Liu W. Li H. (2021). Lignin-based electrodes for energy storage application. *Ind. Crops Prod.* 165:113425. 10.1016/j.indcrop.2021.113425

[B46] Liu K. Du H. Zheng T. Liu H. Zhang M. Zhang R. (2021). Recent advances in cellulose and its derivatives for oilfield applications. *Carbohydr. Polym.* 259:117740. 10.1016/j.carbpol.2021.117740 33674000

[B47] Liu W. Du H. Zhang M. Liu K. Liu H. Xie H. (2020). Bacterial cellulosebased composite scaffolds for biomedical applications: a review. *ACS Sustain. Chem. Eng.* 8 7536–7562. 10.1021/acssuschemeng.0c00125

[B48] Liu X. W. Luan S. Li W. (2019). Utilization of waste hemicelluloses lye for superabsorbent hydrogel synthesis. *Int. J. Biol. Macromol.* 132 954–962. 10.1016/j.ijbiomac.2019.04.041 30974135

[B49] Lucenius J. Valle-Delgado J. J. Parikka K. Osterberg M. (2019). Understanding hemicellulose-cellulose interactions in cellulose nanofibril-based composites. *J. Colloid. Interf. Sci.* 555 104–114. 10.1016/j.jcis.2019.07.053 31377636

[B50] Luo Y. P. Li Z. Li X. L. Liu X. F. Fan J. J. Clark J. H. (2019). The production of furfural directly from hemicellulose in lignocellulosic biomass: a review. *Catal. Today* 319 14–24. 10.1016/j.cattod.2018.06.042

[B51] Ma M. G. Jia N. Zhu J. F. Li S. M. Peng F. Sun R. C. (2012). Isolation and characterization of hemicelluloses extracted by hydrothermal pretreatment. *Bioresour. Technol.* 114 677–683. 10.1016/j.biortech.2012.03.048 22487132

[B52] Ma Q. L. Zhu J. J. Gleisner R. Yang R. D. Zhu J. Y. (2018). Valorization of wheat straw using a recyclable hydrotrope at low temperatures (<= 90 degrees C). *ACS Sustain. Chem. Eng.* 6 14480–14489. 10.1021/acssuschemeng.8b03135

[B53] Manaf S. F. A. Jahim J. M. Harun S. Luthfi A. A. I. (2018). Fractionation of oil palm fronds (OPF) hemicellulose using dilute nitric acid for fermentative production of xylitol. *Ind. Crops Prod.* 115 6–15. 10.1016/j.indcrop.2018.01.067

[B54] Marcotullio G. Krisanti E. Giuntoli J. de Jong W. (2011). Selective production of hemicellulose-derived carbohydrates from wheat straw using dilute HCl or FeCl3 solutions under mild conditions. X-ray and thermo-gravimetric analysis of the solid residues. *Bioresour. Technol.* 102 5917–5923. 10.1016/j.biortech.2011.02.092 21421304

[B55] Middleton R. S. Carey J. W. Currier R. P. Hyman J. D. Kang Q. J. Karra S. (2015). Shale gas and non-aqueous fracturing fluids: opportunities and challenges for supercritical CO2. *Appl. Energy* 147 500–509. 10.1016/j.apenergy.2015.03.023

[B56] Mihiretu G. T. Brodin M. Chimphango A. F. Oyaas K. Hoff B. H. Gorgens J. F. (2017). Single-step microwave-assisted hot water extraction of hemicelluloses from selected lignocellulosic materials - a biorefinery approach. *Bioresour. Technol.* 241 669–680. 10.1016/j.biortech.2017.05.159 28609755

[B57] Mihiretu G. T. Chimphango A. F. Gorgens J. F. (2019). Steam explosion pre-treatment of alkali-impregnated lignocelluloses for hemicelluloses extraction and improved digestibility. *Bioresour. Technol.* 294:122121. 10.1016/j.biortech.2019.122121 31561152

[B58] Mugwagwa L. R. Chimphango A. F. A. (2020). Optimising wheat straw alkali-organosolv pre-treatment to enhance hemicellulose modification and compatibility with reinforcing fillers. *Int. J. Biol. Macromol.* 143 862–872. 10.1016/j.ijbiomac.2019.09.147 31689409

[B59] Nascimento P. F. P. Neto E. L. B. (2021). Steam explosion: hydrothermal pretreatment in the production of an adsorbent material using coconut husk. *BioEnergy Res.* 14 153–162. 10.1007/s12155-020-10159-y

[B60] Nigam J. N. (2001). Ethanol production from wheat straw hemicellulose hydrolysate by Pichia stipitis. *J. Biotechnol.* 87 17–27. 10.1016/S0168-1656(00)00385-0 11267696

[B61] Peng F. Peng P. Xu F. Sun R. C. (2012). Fractional purification and bioconversion of hemicelluloses. *Biotechnol. Adv.* 30 879–903. 10.1016/j.biotechadv.2012.01.018 22306329

[B62] Peng F. Ren J. L. Peng B. Xu F. Sun R. C. Sun J. X. (2008). Rapid homogeneous lauroylation of wheat straw hemicelluloses under mild conditions. *Carbohydr. Res.* 343 2956–2962. 10.1016/j.carres.2008.08.023 18793765

[B63] Peng X. Chen H. (2012). Hemicellulose sugar recovery from steam-exploded wheat straw for microbial oil production. *Process Biochem.* 47 209–215. 10.1016/j.procbio.2011.10.035

[B64] Pereira P. H. F. Waldron K. W. Wilson D. R. Cunha A. P. de Brito E. S. Rodrigues T. H. S. (2017). Wheat straw hemicelluloses added with cellulose nanocrystals and citric acid. effect on film physical properties. *Carbohydr. Polym.* 164 317–324. 10.1016/j.carbpol.2017.02.019 28325332

[B65] Persson T. Ren J. L. Joelsson E. Jonsson A. S. (2009). Fractionation of wheat and barley straw to access high-molecular-mass hemicelluloses prior to ethanol production. *Bioresour. Technol.* 100 3906–3913. 10.1016/j.biortech.2009.02.063 19349171

[B66] Qi G. X. Xiong L. Li H. L. Huang Q. L. Luo M. T. Tian L. L. (2019). Hydrotropic pretreatment on wheat straw for efficient biobutanol production. *Biomass Bioenergy.* 122 76–83. 10.1016/j.biombioe.2019.01.039

[B67] Ragab T. Amer H. Mossa A. T. Emam M. Hasaballah A. A. Helmy W. A. J. B. (2018). Anticoagulation, fibrinolytic and the cytotoxic activities of sulfated hemicellulose extracted from rice straw and husk. *Biocatal. Agric. Biotechnol.* 15 86–91. 10.1016/j.bcab.2018.05.010

[B68] Rao J. Gao H. Guan Y. Li W. Q. Liu Q. (2019). Fabrication of hemicelluloses films with enhanced mechanical properties by graphene oxide for humidity sensing. *Carbohydr. Polym.* 208 513–520. 10.1016/j.carbpol.2018.12.099 30658831

[B69] Relvas F. M. Morais A. R. C. Bogel-Lukasik R. (2015). Selective hydrolysis of wheat straw hemicellulose using high-pressure CO2 as catalyst. *RSC Adv.* 5 73935–73944. 10.1039/C5RA14632A

[B70] Ren J. L. Peng X. W. Zhong L. X. Peng F. Sun R. C. (2012). Novel hydrophobic hemicelluloses: synthesis and characteristic. *Carbohydr. Polym.* 89 152–157. 10.1016/j.carbpol.2012.02.064 24750617

[B71] Ren J. L. Sun R. C. Liu C. F. Cao Z. N. Luo W. (2007). Acetylation of wheat straw hemicelluloses in ionic liquid using iodine as a catalyst. *Carbohydr. Polym.* 70 406–414. 10.1016/j.carbpol.2007.04.022

[B72] Ren J. L. Xu F. Sun R. C. Peng B. Sun J. X. (2008). Studies of the lauroylation of wheat straw hemicelluloses under heating. *J. Agric. Food Chem.* 56 1251–1258. 10.1021/jf072983q 18237136

[B73] Roberto I. C. Sato S. de Mancilha I. M. (1996). Effect of inoculum level of xylitol production from rice straw hemicellulose hydrolysate by Candida guilliermondii. *J. Ind. Microbiol.* 16 348–350. 10.1007/BF01570113 8987492

[B74] Scordia D. Cosentino S. L. Lee J. W. Jeffries T. W. (2012). Bioconversion of giant reed (*Arundo donax* L.) hemicellulose hydrolysate to ethanol by *Scheffersomyces stipitis* CBS6054. *Biomass Bioenergy* 39 296–305. 10.1016/j.biombioe.2012.01.023

[B75] Sharma S. Nandal P. Arora A. (2019). Ethanol production from NaOH pretreated rice straw: a cost effective option to manage rice crop residue. *Waste Biomass Valorization* 10 3427–3434. 10.1007/s12649-018-0360-4

[B76] Shi J. B. Yang Q. L. Lin L. Peng L. C. (2013). Fractionation and characterization of physicochemical and structural features of corn stalk hemicelluloses from yellow liquor of active oxygen cooking. *Ind. Crops Prod.* 44 542–548. 10.1016/j.indcrop.2012.09.026

[B77] Shi Y. F. Du X. H. Jin M. T. Wu S. Wang L. Qiao N. (2021). A two-step process for pre-hydrolysis of hemicellulose in pulp-impregnated effluent with high alkali concentration to improve xylose production. *J. Hazard. Mater.* 402:123573. 10.1016/j.jhazmat.2020.123573 32738785

[B78] Sipponen M. H. Pihlajaniemi V. Sipponen S. Pastinen O. Laakso S. (2014). Autohydrolysis and aqueous ammonia extraction of wheat straw: effect of treatment severity on yield and structure of hemicellulose and lignin. *RSC Adv.* 4 23177–23184. 10.1039/C4RA03236E

[B79] Sun D. Wang H. M. Wang B. Wen J. L. Li M. F. Sun R. C. (2019). Comparative study of hemicelluloses from Hybrid Pennisetum via a green and clean integrated process. *Carbohydr. Polym.* 205 135–142. 10.1016/j.carbpol.2018.10.027 30446088

[B80] Sun R. C. Tomkinson J. (2003). Characterization of hemicelluloses isolated with tetraacetylethylenediamine activated peroxide from ultrasound irradiated and alkali pre-treated wheat straw. *Eur. Polym. J.* 39 751–759. 10.1016/S0014-3057(02)00274-4

[B81] Sun R. C. Fang J. M. Tomkinson J. Chemistry F. (2000). Characterization and esterification of hemicelluloses from rye straw. *J. Agric. Food Chem.* 48 1247–1252. 10.1021/jf990570m 10775380

[B82] Sun R. C. Sun X. F. Ma X. H. (2002). Effect of ultrasound on the structural and physiochemical properties of organosolv soluble hemicelluloses from wheat straw. *Ultrason. Sonochem.* 9 95–101. 10.1016/S1350-4177(01)00102-X 11794024

[B83] Sun S. L. Wen J. L. Ma M. G. Song X. L. Sun R. C. (2014). Integrated biorefinery based on hydrothermal and alkaline treatments: investigation of sorghum hemicelluloses. *Carbohydr. Polym.* 111 663–669. 10.1016/j.carbpol.2014.04.099 25037401

[B84] Sun X. F. Gan Z. Jing Z. X. Wang H. H. Wang D. Jin Y. A. (2015a). Adsorption of methylene blue on hemicellulose-based stimuli-responsive porous hydrogel. *J. Appl. Polym. Sci.* 132:41606. 10.1002/app.41606

[B85] Sun X. F. Jing Z. X. Fowler P. Wu Y. G. Rajaratnam M. (2011). Structural characterization and isolation of lignin and hemicelluloses from barley straw. *Ind. Crop Prod.* 33 588–598. 10.1016/j.indcrop.2010.12.005

[B86] Sun X. F. Liu B. C. Jing Z. X. Wang H. H. (2015b). Preparation and adsorption property of xylan/poly(acrylic acid) magnetic nanocomposite hydrogel adsorbent. *Carbohydr. Polym.* 118 16–23. 10.1016/j.carbpol.2014.11.013 25542101

[B87] Sun X. F. Sun R. C. Fowler P. Baird M. S. (2005a). Extraction and characterization of original lignin and hemicelluloses from wheat straw. *J. Agric. Food Chem.* 53 860–870. 10.1021/jf040456q 15712990

[B88] Sun X. F. Wang H. H. Jing Z. X. Mohanathas R. (2013). Hemicellulose-based pH-sensitive and biodegradable hydrogel for controlled drug delivery. *Carbohydr. Polym.* 92 1357–1366. 10.1016/j.carbpol.2012.10.032 23399165

[B89] Sun X. F. Xu F. Zhao H. Sun R. C. Fowler P. Baird M. S. (2005b). Physicochemical characterisation of residual hemicelluloses isolated with cyanamide-activated hydrogen peroxide from organosolv pre-treated wheat straw. *Bioresour. Technol.* 96 1342–1349. 10.1016/j.biortech.2004.11.018 15792581

[B90] Tabañag I. D. F. Chu I. M. Wei Y. H. Tsai S. L. (2018). Ethanol production from hemicellulose by a consortium of different genetically-modified sacharomyces cerevisiae. *J. Taiwan Inst. Chem. Eng.* 89 15–25. 10.1016/j.jtice.2018.04.029

[B91] Terrone C. C. de Freitas C. Fanchini Terrasan C. R. de Almeida A. F. Carmona E. C. (2018). Agroindustrial biomass for xylanase production by *Penicillium chrysogenum*: purification, biochemical properties and hydrolysis of hemicelluloses. *Electron. J. Biotechnol.* 33 39–45. 10.1016/j.ejbt.2018.04.001

[B92] Thomsen M. H. Thygesen A. Thomsen A. B. (2008). Hydrothermal treatment of wheat straw at pilot plant scale using a three-step reactor system aiming at high hemicellulose recovery, high cellulose digestibility and low lignin hydrolysis. *Bioresour. Technol.* 99 4221–4228. 10.1016/j.biortech.2007.08.054 17936621

[B93] Thuvander J. Jönsson A. S. (2019). Influence of air and nitrogen sparging on flux during ultrafiltration of hemicelluloses extracted from wheat bran. *Sep. Purif. Technol.* 212 84–88. 10.1016/j.seppur.2018.11.010

[B94] Thuvander J. Jonsson A. S. (2020). Techno-economic impact of air sparging prior to purification of alkaline extracted wheat bran hemicelluloses by membrane filtration. *Sep. Purif. Technol.* 253:117498. 10.1016/j.seppur.2020.117498

[B95] Thuvander J. Arkell A. Jonsson A. S. (2018). Reduction of energy demand by use of air sparging during ultrafiltration of alkali-extracted wheat bran hemicelluloses. *Chem. Eng. Res. Des.* 138 43–50. 10.1016/j.cherd.2018.08.001

[B96] Tian S. Q. Zhao R. Y. Chen Z. C. (2018). Review of the pretreatment and bioconversion of lignocellulosic biomass from wheat straw materials. *Sustainable Energy Rev.* 91 483–489. 10.1016/j.rser.2018.03.113

[B97] Tsegaye B. Balomajumder C. Roy P. (2019). Optimization of microwave and NaOH pretreatments of wheat straw for enhancing biofuel yield. *Energy Convers. Manag.* 186 82–92. 10.1016/j.enconman.2019.02.049

[B98] Tsegaye B. Balomajumder C. Roy P. (2020). Organosolv pretreatments of rice straw followed by microbial hydrolysis for efficient biofuel production. *Renewable Energy* 148 923–934. 10.1016/j.renene.2019.10.176

[B99] Wang R. Z. Yue J. F. Jiang J. C. Li J. Zhao J. P. Xia H. H. (2021). Hydrothermal CO2-assisted pretreatment of wheat straw for hemicellulose degradation followed with enzymatic hydrolysis for glucose production. *Waste Biomass Valorization* 12 1483–1492. 10.1007/s12649-020-01103-4

[B100] Wang T. Li C. Song M. Grain R. F. J. (2019). Xylo-oligosaccharides preparation through acid hydrolysis of hemicelluloses isolated from press-lye. *Grain Oil Sci. Technol.* 2 21–25. 10.1016/j.gaost.2019.07.001

[B101] Xu F. Jiang J. X. Sun R. C. She D. Peng B. Sun J. X. (2008). Rapid esterification of wheat straw hemicelluloses induced by microwave irradiation. *Carbohydr. Polym.* 73 612–620. 10.1016/j.carbpol.2008.01.002 26048228

[B102] Xu F. Liu C. F. Geng Z. C. Sun J. X. Sun R. C. Hei B. H. (2006). Characterisation of degraded organoslv hemicelluloses from wheat straw. *Polym. Degrad. Stabil.* 91 1880–1886. 10.1016/j.polymdegradstab.2005.11.002

[B103] Yang H. P. Li S. J. Liu B. Chen Y. Q. Xiao J. J. Dong Z. G. (2020). Hemicellulose pyrolysis mechanism based on functional group evolutions by two-dimensional perturbation correlation infrared spectroscopy. *Fuel* 267:117302. 10.1016/j.fuel.2020.117302

[B104] Yang H. Q. Yi N. Zhao S. Qaseem M. F. Zheng B. Li H. L. (2020). Characterization of hemicelluloses in sugarcane (Saccharum spp. hybrids) culm during xylogenesis. *Int. J. Biol. Macromol.* 165 1119–1128. 10.1016/j.ijbiomac.2020.09.242 33035529

[B105] Yao S. Q. Nie S. X. Zhu H. X. Wang S. F. Song X. P. Qin C. R. (2017). Extraction of hemicellulose by hot water to reduce adsorbable organic halogen formation in chlorine dioxide bleaching of bagasse pulp. *Ind. Crop Prod.* 96 178–185. 10.1016/j.indcrop.2016.11.046

[B106] Yoon K. Y. Woodams E. E. Hang Y. D. (2006). Enzymatic production of pentoses from the hemicellulose fraction of corn residues. *LWT Food Sci. Technol.* 39 388–392. 10.1016/j.lwt.2005.02.005

[B107] You X. Wang X. Liang C. Liu X. L. Wang S. F. (2019). Purification of hemicellulose from sugarcane bagasse alkaline hydrolysate using an aromatic-selective adsorption resin. *Carbohydr. Polym.* 225:115216. 10.1016/j.carbpol.2019.115216 31521307

[B108] Yuan Y. Zou P. Zhou J. H. Geng Y. T. Fan J. J. Clark J. (2019). Microwave-assisted hydrothermal extraction of non-structural carbohydrates and hemicelluloses from tobacco biomass. *Carbohydr. Polym.* 223:11543. 10.1016/j.carbpol.2019.115043 31426995

[B109] Zhang J. Wang Y. H. Qu Y. S. Wei Q. Y. Li H. Q. (2018). Effect of the organizational difference of corn stalk on hemicellulose extraction and enzymatic hydrolysis. *Ind. Crops Prod.* 112 698–704. 10.1016/j.indcrop.2018.01.007

[B110] Zhang W. Zhu S. Bai Y. Xi N. Wang S. Bian Y. (2015). Glow discharge electrolysis plasma initiated preparation of temperature/pH dual sensitivity reed hemicellulose-based hydrogels. *Carbohydr. Polym.* 122 11–17. 10.1016/j.carbpol.2015.01.007 25817637

[B111] Zhao W. Glavas L. Odelius K. Edlund U. Albertsson A. C. (2014). Facile and green approach towards electrically conductive hemicellulose hydrogels with tunable conductivity and swelling behavior. *Chem. Mater.* 26 4265–4273. 10.1021/cm501852w

[B112] Zhao Y. L. Sun H. Yang B. Weng Y. X. (2020). Hemicellulose-based film: potential green films for food packaging. *Polymers* 12:1775. 10.3390/polym12081775 32784786 PMC7465936

[B113] Zhong C. Wang C. Huang F. Wang F. Jia H. Zhou H. (2015). Selective hydrolysis of hemicellulose from wheat straw by a nanoscale solid acid catalyst. *Carbohydr. Polym.* 131 384–391. 10.1016/j.carbpol.2015.05.070 26256198

